# Intervention Approaches in Studying the Response to Vitamin D_3_ Supplementation

**DOI:** 10.3390/nu15153382

**Published:** 2023-07-29

**Authors:** Emilia Gospodarska, Ranjini Ghosh Dastidar, Carsten Carlberg

**Affiliations:** 1 Institute of Animal Reproduction and Food Research, Polish Academy of Sciences, PL-10-748 Olsztyn, Poland; e.gospodarska@pan.olsztyn.pl (E.G.); r.dastidar@pan.olsztyn.pl (R.G.D.); 2 School of Medicine, Institute of Biomedicine, University of Eastern Finland, FI-70211 Kuopio, Finland

**Keywords:** vitamin D, vitamin D intervention trials, vitamin D response index, transcriptome analysis, epigenome analysis, modeling, N = 1

## Abstract

Vitamin D intervention studies are designed to evaluate the impact of the micronutrient vitamin D_3_ on health and disease. The appropriate design of studies is essential for their quality, successful execution, and interpretation. Randomized controlled trials (RCTs) are considered the “gold standard” for intervention studies. However, the most recent large-scale (up to 25,000 participants), long-term RCTs involving vitamin D_3_ did not provide any statistically significant primary results. This may be because they are designed similarly to RCTs of a therapeutic drug but not of a nutritional compound and that only a limited set of parameters per individual were determined. We propose an alternative concept using the segregation of study participants into different groups of responsiveness to vitamin D_3_ supplementation and in parallel measuring a larger set of genome-wide parameters over multiple time points. This is in accordance with recently developed mechanistic modeling approaches that do not require a large number of study participants, as in the case of statistical modeling of the results of a RCT. Our experience is based on the vitamin D intervention trials VitDmet, VitDbol, and VitDHiD, which allowed us to distinguish the study participants into high, mid, and low vitamin D responders. In particular, investigating the vulnerable group of low vitamin D responders will provide future studies with more conclusive results both on the clinical and molecular benefits of vitamin D_3_ supplementation. In conclusion, our approach suggests a paradigm shift towards detailed investigations of transcriptome and epigenome-wide parameters of a limited set of individuals, who, due to a longitudinal design, can act as their own controls.

## 1. Introduction

Vitamin D_3_ was identified more than 100 years ago as the essential molecule preventing the bone malformation disorder rickets and was therefore termed a vitamin [1]. Since vitamin D_3_ can be synthesized endogenously in UV-B exposed skin [2,3], the need for this vitamin classification is in part due to human migration from Africa to Northern latitudes in Europe, Asia and America over the past 50,000 years [4]. Above a latitude of 38° N during winter, there is a period of 1–5 months in which no or insufficient amounts of UV-B reach the surface for vitamin D_3_ synthesis. However, over many thousands of years hunter–gatherer populations adapted genetically to reduced UV-B exposure, such as a less active DHCR7 (7-dehydrocholesterol reductase) enzyme [5]. Reduced activity of DHCR7 results in higher concentrations of 7-dehydrocholesterol in the skin so that even less intense UV-B exposure can lead to sufficient conversion of the cholesterol precursor into vitamin D_3_ [6,7]. In parallel, genetic polymorphisms causing reduced activity of enzymes and transporters such as those encoded by the genes *SLC24A5* (solute carrier family 24 member 5) and *SLC45A2* of the solute carrier family [7,8] involved in melanin synthesis resulted in lighter skin of Siberian and European populations [9]. In addition, some populations living close to the coast benefitted from adding vitamin D_3_-rich food like fatty fish to their diet [10,11]. Although vitamin D deficiency caused bone malformations already in developed ancient societies, such as in the Roman Empire [12,13], just since the times of the Industrial Revolution, which was characterized by working and living conditions with very reduced sun exposure, vitamin D_3_ became a real vitamin. For example, in England in the 19th century, rickets was a very common disorder in children [14,15]. This became even more severe with the worldwide preference for indoor activity and coverage with textile outdoors. Thus, vitamin D deficiency is nowadays a global problem [16].

The vitamin D status of an individual is defined as the serum concentration of the most stable and abundant vitamin D metabolite, 25-hydroxyvitamin D_3_ (25(OH)D_3_) [17]. There is an ongoing debate about the recommended vitamin D status, but most researchers agree that it should be in the range of 75–100 nM (30–40 ng/mL) 25(OH)D_3_ [18]. As a reference, the vitamin D status of a population having a lifestyle rather close to ancient hunter–gatherers, the Hadza tribe in Tanzania, is on average 110 nM [19]. This suggests that nowadays we should aim for a comparable level. Accordingly, a vitamin D status of less than 50 nM (20 ng/mL) 25(OH)D_3_ is considered insufficient [20], because it significantly increases the risk for musculoskeletal disorders [21], and a level below 30 nM (12 ng/mL) is defined as vitamin D deficiency [22,23]. While the musculoskeletal benefits of a sufficient vitamin D status are well established, the role of vitamin D in non-skeletal tissues such as the immune system and related diseases, such as type I diabetes [24], multiple sclerosis [25,26] and inflammatory bowel disease [27] as well as the prevention of severe consequences from infections with SARS-CoV2, influenza [28,29] or the intracellular bacterium *Mycobacterium tuberculosis* [30,31], still needs to be further evaluated. Furthermore, there are indications that vitamin D has also a protective role against the development of several cancers, such as colon, breast, and prostate [32], as well as type 2 diabetes and cardiovascular diseases [33]. However, these protective effects are likely an indirect consequence of the immune regulatory function of vitamin D [34]. To evaluate the possible pleiotropic clinical benefit of vitamin D, some 10 years ago, several large-scale vitamin D intervention studies have been initiated.

The physiological effects of vitamin D are mediated by the biologically most active form of vitamin D_3_, 1,25-dihydroxyvitamin D_3_ (1,25(OH)_2_D_3_), which acts as a high-affinity ligand to the transcription factor VDR (vitamin D receptor) [35]. In this way, vitamin D controls the transcription of hundreds of target genes in *VDR*-expressing tissues and cell types [36]. Thus, a part of the human transcriptome is responsive to vitamin D. A prerequisite to the modulation of the transcriptome is changes in the epigenome of vitamin D target cell types. These comprise the ligand-triggered (i) binding of VDR to genomic DNA [37], (ii) association of VDR with helping proteins such as pioneer factors [38], (iii) modulation of post-translational histone modifications [39], and (iv) changes in chromatin accessibility [40]. In this perspective, vitamin D_3_ intervention studies can be considered as nutrigenomic experiments, in which the action of vitamin D_3_ and its endogenously produced metabolites are investigated under human in vivo conditions. This had already been demonstrated by studying vitamin D-triggered changes in chromatin accessibility [41] and target gene regulation [42].

In this review, we first discuss why the primary analysis of the results of RCTs could not show statistically significant effects on most of the expected endpoints. Furthermore, we propose an alternative approach, the core of which is the segregation of study participants into high, mid, and low vitamin D responders. Proper vitamin D_3_ supplementation of low vitamin D responders and longitudinally measuring their molecular response on the level of the epigenome and transcriptome may provide more conclusive results concerning the benefits of vitamin D_3_ than large-scale RCTs and, in addition, allow a molecular understanding of the actions of vitamin D in a human in vivo setting.

## 2. Large-Scale Vitamin D Intervention Studies

RCTs aim to measure the effectiveness of interventions or treatments, such as whether vitamin D_3_ supplementation improves a disease condition or its onset. The studies are randomized, i.e., the participants are randomly assigned to a group that is receiving treatment or not. RCTs are often blinded so that neither researchers nor participants know who is receiving a treatment and who is not. This reduces the bias from conclusions about the study results concerning the relation of an intervention and its outcome, i.e., possible confounding effects (known as well as unknown) are balanced. This clearly distinguishes RCTs from observational studies. However, the design of RCTs requires a larger number of participants. Finally, when the RCTs are finished, they are unblinded and mostly analyzed in comparison of the groups to which the individuals had been assigned, i.e., treatment versus placebo.

Within the past 6 years, the results of a few large-scale RCTs involving vitamin D_3_ supplementation have been published (Table 1). The largest study with more than 25,000 participants is VITAL (VITamin D and OmegA-3 TriaL), which investigated, over a period of more than 5 years, the effect of daily supplementation with 50 µg (2000 IU) vitamin D_3_ and/or 1 g ω-3 fatty acids concerning the prevention of cardiovascular disease and cancer [43]. The ViDA (Vitamin D Assessment) study tested monthly vitamin D_3_ bolus supplementations (100,000 IU) with more than 5000 adults over 3.3 years concerning the prevention of cardiovascular events and mortality [44]. In the FIND (FINnish Vitamin D) trial, 2500 older individuals were treated with 40 or 80 µg vitamin D_3_ (1600 or 3200 IU) per day over 5 years for investigation of the primary outcomes of cardiovascular disease and invasive cancer [45]. The study D2d (vitamin D and type 2 diabetes) included nearly 2500 subjects, which received daily 100 µg vitamin D_3_ (4000 IU) over 2.5 years, for a possible conversion of prediabetes to type 2 diabetes (T2D) [46]. Finally, the DO-HEALTH trial investigated, with more than 2000 participants, whether a daily vitamin D_3_ dose of 50 µg (2000 IU) alone or in combination with ω-3 (1 g/d) and a strength-training exercise program over 3 years improves the health conditions of elderly, such as the likelihood of falls [47].

The average vitamin D status of a total of 38,054 individuals in these five studies was, at baseline, more than 50 nM 25(OH)D_3_ in serum, i.e., hardly any study participants were vitamin D deficient. This is not representative of the world population, of which some 7% are severely vitamin D deficient and 33% have an insufficient vitamin D status [48]. Moreover, the average age of the subjects was between 60 and 75 years, i.e., the focus was on the elderly (Table 1). In all five studies, vitamin D_3_ supplementation clearly increased the vitamin D status of the participants to sufficiency. However, primary analysis of the data showed a trend in the expected direction, but did not provide any statistically significant indication that vitamin D_3_ supplementation, either daily or monthly, was beneficial for the expected clinical outcome of the respective trials, i.e., the studies had null results [23].

There are several explanations for this unexpected result. The main point may be that the study participants were recruited an excessively high basal vitamin D status, i.e., many of them were already vitamin D sufficient and further vitamin D_3_ supplementation did not improve their clinical status (only 12.7% of participants in VITAL and 9.1% in FIND were vitamin D deficient). In the classical RCT, the entrance criterion into a study should be low vitamin D_3_ status to observe a stronger effect of supplementation, but for ethical reasons, long-term RCTs with vitamin-D_3_-deficient individuals as a placebo group (control) cannot be performed. Additionally, for some of the individuals, daily supplementation with only 40 µg (FIND) or 50 µg (VITAL and DO-HEALTH) vitamin D_3_ may have been insufficient. Furthermore, the RCTs were designed for outcomes such as cancer, cardiovascular disease, and T2D that do not reflect the primary physiological role of vitamin D, which is the control of calcium homeostasis and the modulation of immunity [49]. For example, secondary analysis of VITAL results indicated that vitamin D_3_ supplementation reduced the risk of autoimmune diseases by 22%, i.e., for a directly immune-related outcome a significant effect could be observed [50]. An additional option is the use of specific subgroups, such as the exclusive recruitment of normal-weight persons (body mass index < 25). Secondary analysis of the results of the five large trials indicated significant results for normal-weight persons and/or individuals with vitamin D deficiency at baseline. Moreover, VITAL indicated there were long-term benefits of vitamin D_3_ supplementation on cancer mortality [51], while ViDA was too short-term to confirming this result, i.e., the duration of RCTs is an important parameter. Thus, there are several options to improve the design of RCTs involving vitamin D_3_.

## 3. Genome-Wide Analyses

**Figure 1:**
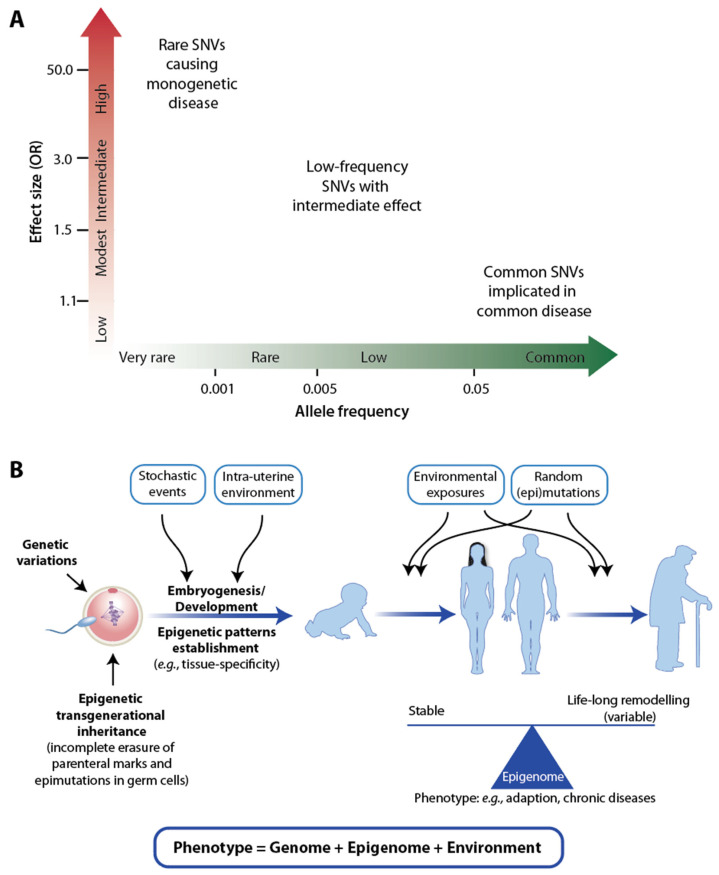
Genome, epigenome, and environment. A graph of the strength of a genetic effect (OR) over risk allele frequency indicates different groups of SNVs (**A**). Genetic and epigenetic effects throughout life from “womb to tomb” (**B**). More details are provided in the text.

“Big biology” projects such as 1000 Genomes (www.internationalgenome.org, accessed on 5 July 2023) have described the variability of the human genome [52]. On average, two individuals will differ by 4–5 million SNVs (single nucleotide variants) and some 1000 CNVs (copy number variants). Since some 20 years ago, a huge number of genome-wide association studies (GWAS) have been performed to find statistically significant associations between SNVs and various traits [53]. These traits can be anthropomorphic properties such as height or skin color but also the susceptibility to diseases. For basically all common diseases, dozens to hundreds of SNVs were identified, but nearly all showed only a minor contribution, expressed by an odds ratio (OR) in the order of 5–20% increased or decreased disease risk, while monogenetic diseases are based on one dominant variation with very high ORs (Figure 1A). The genetic composition of all cells within a body is determined at the moment of conception and will stay the same assuming the person does not develop cancer (Figure 1B). Thus, the genetic contribution to our lives cannot be influenced. In this context, it is important that, despite large efforts, GWAS results can predict only some 20% of traits on the basis of genetic variations. Although there is still the possibility that some of the missing heritability may be explained by rare, yet unidentified SNVs with medium ORs, most of the remaining 80% are related to environmental exposure and epigenetics (Figure 1B). This finding implies that a large part of our individual disease risk is modulated by environmental triggers, which are majorly influenced by our lifestyle decisions. Thus, environmental exposures, including those that we experience as fetuses, affect the epigenome and may explain large parts of the missing heritability. This insight can be summarized by the formula “phenotype = genetics + epigenetics + environment” (Figure 1B) and implies that we are, to a large extent, individually responsible for staying healthy and avoiding major common diseases.

The 20/80% contribution of genetics versus environment/epigenetics also applies to vitamin D status. GWAS indicated that SNVs in the regions of the genes *DHCR7* (rs12785878, rs7940244, and rs7944926), *CYP2R1* (cytochrome P450 family 2 subfamily R member 1, rs10741657), *CYP24A1* (rs17216707), and *GC* (GC vitamin D-binding protein, rs3755967) contribute to basal serum levels of 25(OH)D_3_, but each of them only with a small OR [54,55]. Moreover, the derived allele rs12785878 reduces the expression of the *DHCR7* gene, increases the 7-dehydrocholesterol concentrations in the skin, and leads to a more efficient synthesis of vitamin D_3_ [56]. Today’s Europeans have a 2.4-to 3.1-fold higher frequency of the derived allele of rs12785878 than African and Asian populations, i.e., they can better manage low UV-B exposure and still synthesize sufficient vitamin D_3_. Similarly, the frequency of other SNVs related to genes mediating vitamin D signaling, such as *VDR* (rs2228570 (known as *FokI* polymorphism), rs1544410 (*Bsm1*), rs731236 (*Taq1*)), and the VDR target genes *CD14* (rs2569190) and *CARD9* (caspase recruitment domain family member 9, rs4077515) is increased in European populations [9]. Due to positive evolutionary selection, Europeans appear to be more sensitive to vitamin D than populations from Asia or Africa. The total number of SNVs influencing vitamin D status and vitamin D signaling is nearly 150, but, in total, they predict only a smaller proportion of the trait [55]. Interestingly, a large twin study demonstrated that skin color and sun exposure behavior are major contributors to 25(OH)D_3_ serum levels [57]. This emphasizes that vitamin D status is also a trait that is primarily modulated by epigenetics and environment.

## 4. Segregation of Participants of Vitamin D Intervention Studies

**Figure 2:**
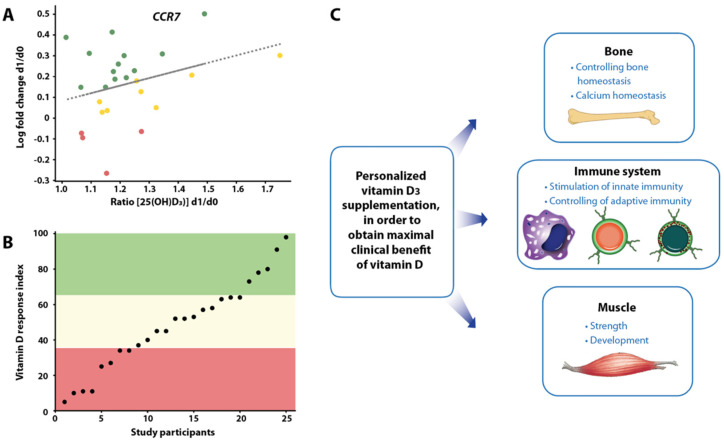
Principles of vitamin D response index determination. Participants of a vitamin D intervention study were classified as high (green), mid (yellow), and low responders (red) to vitamin D_3_ supplementation (here shown in the example of the expression of the *CCR7* (C-C motif chemokine receptor 7) gene, (**A**)). The vitamin D response index is determined by *k-means* ranking of the sum of the scores of a larger number of vitamin D target genes (**B**). Determining the vitamin D response index of an individual will allow personalized supplementation with vitamin D_3_ in order to obtain optimal clinical benefits, such as prevention of osteoporosis, sarcopenia, and autoimmune diseases (**C**).

The small-scale vitamin D intervention studies VitDmet (NCT01479933, ClinicalTrials.gov, accessed on 5 July 2023) [58,59,60,61] and VitDbol (NCT02063334) [62,63] were designed as medical experiments rather than as RCTs (Table 2). VitDmet is a three-arm trial where 71 elderly pre-diabetic subjects were supplemented daily with either 0, 40, or 80 µg vitamin D_3_ over 5 months of a Finnish winter (i.e., no endogenous vitamin D_3_ production possible), i.e., it follows the treatment protocol of the FIND trial. The study aimed at preventing the onset of type 2 diabetes; blood samples were collected at the beginning and end of the intervention. In contrast, in VitDbol 35 young healthy subjects were exposed only once to a vitamin D_3_ bolus (2000 µg = 80,000 IU) and samples were taken at days 0, 1, 2, and 30. Importantly, the analysis of both VitDmet and VitDbol differed from other vitamin D intervention studies by relating the changes of vitamin D-triggered parameters, such as vitamin D target gene expression in PBMCs (peripheral blood mononuclear cells), which had been isolated at the end and beginning of the study, to the ratio of the vitamin D status at the respective time points (Figure 2A). Accordingly, the responsiveness of the respective parameters was determined for each study participant in a way that is comparable to the analysis of in vitro dose–response studies [64].

The method of data analysis of these smaller-scale studies is essential for obtaining significant effects of vitamin D_3_ supplementation. For example, the study BEST-D had a similar three-arm design as VitDmet, also focused on the elderly, and measured the expression of cytokines [65]. However, classical analysis of the data led to a null result. For the analyses of VitDmet and VitDbol, the study participants were scored on each tested parameter as no, weak, or strong responders (Figure 2B), i.e., the individuals showed a personalized molecular response to vitamin D_3_ supplementation [66] (Figure 2C). The segregation of the VitDmet participants into high, mid, and low vitamin D responders was based on the accumulated score of 36 vitamin D-triggered molecular parameters, while for VitDbol only 12 factors were used. Interestingly, both studies agreed that 25% of the participants were low vitamin D responders [67].

The vitamin D intervention study VitDHiD (NCT03537027) followed the VitDbol approach in design, but studied 25 participants on the level of their transcriptome [68] (Table 2). Moreover, in vivo results of VitDHiD were backed up by in vitro assays using PBMCs of the same individuals and the biologically active form of vitamin D_3_, 1,25-dihydroxyvitamin D_3_ (1,25(OH)_2_D_3_). In addition, this study included two different approaches, which were a traditional cohort analysis built on single repeats of 25 individuals and a personalized analysis based on testing a limited number of selected participants in triplicate [68]. This led to the discovery that a large number of vitamin D target genes responded in an individual-specific way, i.e., in some persons, these genes were lower or not responsive compared with others. Accordingly, interindividual differences in the vitamin D response index can be explained, at least in part, by person-specific sets of vitamin D target genes. Interestingly, vitamin D target genes differ in their EC_50_-value of 1,25(OH)_2_D_3_ stimulation [69]. In human PBMCs, some genes already show a response at 0.1 nM, while other genes require concentrations of 1 nM and higher. Since vitamin-D-triggered gene expression is based on epigenetics (Section 1), the different sensitivity of target genes suggests that interindividual differences in the vitamin D response index are also based, at least to some extent, on variations of the epigenome.

The molecular basis of the vitamin D response index needs to be explored further. In analogy to the anti-coagulant drug warfarin, as the interindividual difference in the response to it is determined by SNVs in the genes *VKORC1* (vitamin K epoxide reductase complex subunit 1) and *CYP2C9* [70], the vitamin D response index may be primarily explained by genetic variants. However, as already discussed for the vitamin D status (Section 3), genetics has limited potential in explaining this trait. Therefore, it is likely that this also applies to the vitamin D response index, i.e., epigenetics and environment rather than genetics are the molecular basis of this trait. Importantly, the vitamin D response index is independent of the vitamin D status of the individual, i.e., there are high responders with a low vitamin D status and low responders with a high vitamin D status. High responders can handle a low vitamin D status, while low responders need to have a high vitamin D status to benefit from vitamin D.

In general, the transcriptome-wide analysis has an advantage in that, in contrast to the set of 24 vitamin D target genes, which had been selected in the context of the VitDmet study [58,59,60,61], data from several hundred genes, which significantly respond to vitamin D_3_ supplementation, can be used [68]. This significantly increases the accuracy of the response index calculations. Nevertheless, the ultimate goal is to identify a limited set of vitamin D target genes as biomarkers for determining the response index.

## 5. Future View on Vitamin D Intervention Studies

A low vitamin D status most likely occurs in the winter season, in particular in the about 15% of the world’s population living above a latitude of 38° N [71,72], i.e., when there is low or no endogenous vitamin D_3_ production. High vitamin D responders better tolerate these conditions and should suffer less frequently from autoimmune diseases [73], infections [74], and/or cancer [75] because vitamin D contributes to the prevention of these diseases. In contrast, low vitamin D responders represent a vulnerable part of society that requires higher vitamin D_3_ supplementation doses than suggested by population-based recommendations and guidelines that may serve primarily mid vitamin D responders. However, daily vitamin D_3_ supplementation should not be higher than 4000 IU (100 µg) in order to prevent overdose in high vitamin D responders. The most appropriate supplementation may be 1 µg (40 IU)/kg body mass to account for obese individuals.

It is possible that due to the evolutionary adaptation of the European populations to changing environmental conditions, such as the northern migration after the end of the ice age, there are more high vitamin D responders in Northern Europe than in Southern Europe. Since populations in Nordic countries have a higher rate of ancestry from Caucasian pastoralists (called Yamnaya) and Siberian hunter–gatherers than those from the South [76,77], interbreeding within these populations may have conferred high vitamin D sensitivity to Europe.

**Figure 3:**
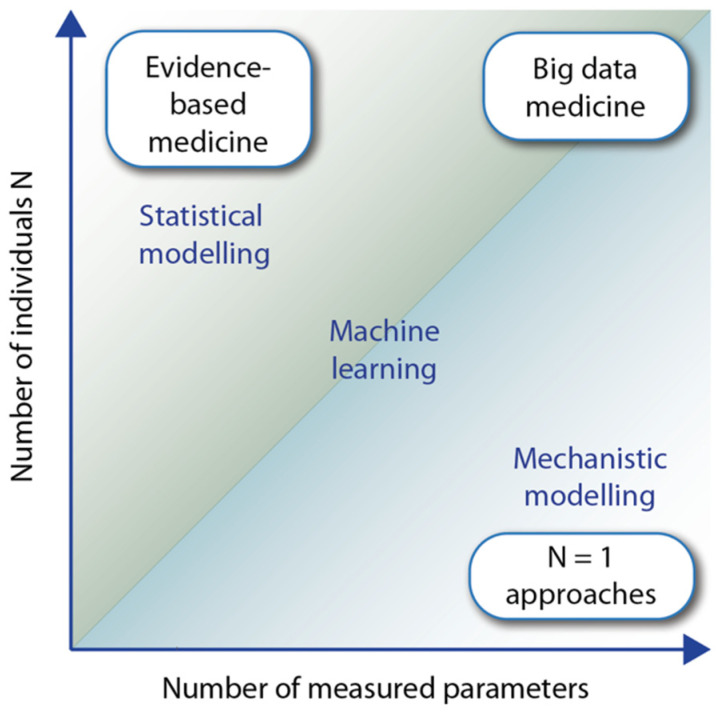
Evidence-based medicine versus N = 1 approaches. The graph displays the number of individuals participating in a clinical study versus the number of variables measured for each individual. In classical evidence-based medicine, many cases and controls are studied for a small number of variables (**top left**), whereas in individual-focused studies (N = 1 approaches), there are only a limited number of people for which many variables are measured longitudinally (**bottom right**). The latter often uses mechanistic modeling for analyzing data, while evidence-based medicine largely relies on statistical modeling. Big data system medicine (**top right**) fuses both approaches by considering many patients and a large number of data points per individual.

Evidence-based medicine aims to enable optimized medical decisions through the integration of a clinician’s experience with personalized data from concerned patients and background knowledge of the respective disease [78,79]. The latter information often derives from RCTs that had been performed with large numbers of cases and controls (Figure 3, top left). The group of low vitamin D responders is assumed to respond more prominently to sufficiently high vitamin D_3_ supplementation than mid or high responders, which already may be saturated with a lower vitamin D status. Accordingly, RCTs performed exclusively with accurately adjusted dosage on low vitamin D_3_ responders would provide more conclusive results. However, ethical considerations do not allow the use of low vitamin D responders as a control group with no or insufficient vitamin D_3_ supplementation. This prevents the design of classical RCTs with low vitamin D responders as well as studies with vitamin-D-deficient persons. In this respect, a longitudinal design of vitamin D intervention studies, such as in VitDmet, VitDbol, or VitDHiD, where each participant is serving as his/her own control, are more appropriate. Since in these studies each participant is investigated individually, they are also referred to as N = 1 studies (Figure 3, bottom right).

The results of classical RCTs are mostly analyzed via statistical models, i.e., through the quantification of mathematical relationships of the measured variables of the study participants. However, this approach cannot be used with a low number of cases, such as in N = 1 approaches. In particular, when the latter approaches measure multi-omic data, such as genome-wide DNA methylation, histone modifications, gene expression, and mechanistic modeling can be applied. In this method, information on biochemical and regulatory pathways, which is obtained from public databases, such as KEGG (Kyoto Encyclopedia of Genes and Genomes) [80], Wikipathways [81], and SPOKE (Scalable Precision Medicine Open Knowledge Engine) [82], can then be used to construct multi-level dynamical computational models [83,84]. Thus, not only the design but also the analysis methods differ clearly between RCTs and N = 1 approaches.

## 6. Conclusions

The most recent large-scale, long-term RCTs involving vitamin D did not provide any significant primary results. This is largely because they were designed similarly to RCTs of therapeutical compounds, i.e., drugs that do not naturally occur in the human body. Thus, for endogenous molecules, such as vitamin D_3_, for which no clean zero controls exist, long-term RCTs with vitamin-D_3_-deficient individuals cannot be performed for ethical reasons and alternative study designs, such as N = 1 approaches, may be more appropriate. Finnish studies such as VitDbol and VitDHiD were designed to use a vitamin D_3_ bolus (Table 2), i.e., they obtained results faster than trials using daily supplementation. The same design was also applied in a vitamin D_3_ intervention study in Saudi Arabia [85,86]. However, a vitamin D_3_ bolus should not be used over longer periods in order to prevent hypercalcemia and tissue calcification [87]. Thus, daily vitamin D_3_ supplementation is recommended [88].

A low responsiveness to vitamin D_3_ supplementation may serve as a trait that identifies members of the general population who have a significantly higher susceptibility to multiple types of diseases, such as autoimmune diseases, cardiovascular disorders, T2D, and cancer. In contrast, high responsiveness may reflect a high immune resilience, i.e., an appropriate response of the immune system to various health challenges [89]. In this way, a low vitamin D index may serve as a warning for generally increased disease susceptibility. However, when future studies are able to validate our assumption that the vitamin D response index is largely determined by epigenetics, concerned individuals will have the chance to reduce their disease risk by better adapting their lifestyle to their given environmental conditions. This may include increased outdoor physical activity paired with exposure to sunlight from early childhood as well as optimized vitamin D_3_ supplementation.

## Figures and Tables

**Table 1:** Major RCTs on vitamin D published within the past 6 years.

Study	Number of Participants	Age (Mean)	25(OH)D_3_ Level Baseline Final		Duration	Intervention (Vitamin D_3_ Dose)
VITAL (USA)	25,871	67 y	77 nM	105 nM	5.3 y	50 µg/day
ViDA (New Zealand)	5108	66 y	66 nM	135 nM	3.3 y	5000 µg once + 2500 µg/month
FIND (Finland)	2495	68 y	75 nM	110 nM	5 y	40 and 80 µg/day
D2d (USA)	2423	60 y	70 nM	135 nM	2.5 y	100 µg/day
DO-HEALTH (Europe)	2157	75 y	56 nM	94 nM	3 y	50 µg /day

**Table 2:** Small-scale studies determining the vitamin D response index.

Study	Number of Participants	Age (Mean)	25(OH)D_3_ Level Baseline Final		Duration	Intervention (Vitamin D_3_ Dose)
VitDmet (Finland)	71	67 y	59 nM	83 nM	5 months	0, 40 or 80 µg/day
VitDbol (Finland)	35	26 y	65 nM	80 nM	30 days	2000 µg bolus once
VitDHiD (Finland)	40	27 y	73 nM	98 nM	1 day	2000 µg bolus once
